# Corticotherapy for traumatic brain-injured Patients - The Corti-TC trial: study protocol for a randomized controlled trial

**DOI:** 10.1186/1745-6215-12-228

**Published:** 2011-10-14

**Authors:** Karim Asehnoune, Antoine Roquilly, Véronique Sebille, The Corti-TC trial group

**Affiliations:** 1 Centre Hospitalier Universitaire de Nantes, Service d'anesthésie réanimation chirurgicale, Hôtel Dieu-HME, Nantes, France; 2 Intensive care Unit, Hotel Dieu, (Place Ricordeau), Nantes (44093), France; 3 Biostatistics Unit. EA 4275, Pharmacy University of Nantes, Nantes F-44000 France; 4 The Corti-TC trial group

**Keywords:** Trauma, Traumatic brain injury, corticosteroid insufficiency, glucocorticoid, mineralocorticoid, pneumonia, nosocomial infection, intensive care unit

## Abstract

**Background:**

Traumatic brain injury (TBI) is a main cause of severe prolonged disability of young patients. Hospital acquired pneumonia (HAP) add to the morbidity and mortality of traumatic brain-injured patients. In one study, hydrocortisone for treatment of traumatic-induced corticosteroid insufficiency (CI) in multiple injured patients has prevented HAP, particularly in the sub-group of patients with severe TBI. Fludrocortisone is recommended in severe brain-injured patients suffering from acute subarachnoid hemorrhage. Whether an association of hydrocortisone with fludrocortisone protects from HAP and improves neurological recovery is uncertain. The aim of the current study is to compare corticotherapy to placebo for TBI patients with CI.

**Methods:**

The CORTI-TC (Corticotherapy in traumatic brain-injured patients) trial is a multicenter, randomized, placebo controlled, double-blind, two-arms study. Three hundred and seventy six patients hospitalized in Intensive Care Unit with a severe traumatic brain injury (Glasgow Coma Scale ≤ 8) are randomized in the first 24 hours following trauma to hydrocortisone (200 mg.day^-1 ^for 7 days, 100 mg on days 8-9 and 50 mg on day-10) with fludrocortisone (50 μg for 10 days) or double placebo. The treatment is stopped if patients have an appropriate adrenal response. The primary endpoint is HAP on day-28. The endpoint of the ancillary study is the neurological status on 6 and 12 months.

**Discussion:**

The CORTI-TC trial is the first randomized controlled trial powered to investigate whether hydrocortisone with fludrocortisone in TBI patients with CI prevent HAP and improve long term recovery.

**Trial registration:**

NCT01093261

## Background

Traumatic brain injury (TBI) is a leading cause of prolonged disability in young patients. The rate of hospital acquired pneumonia (HAP) varies from 30 to 50% [1-3]. Post traumatic HAP increases the risk of intracranial hypertension [4], prolongs duration of mechanical ventilation and Intensive Care Unit (ICU) length of stay [5,6] and may increase the rate of death [7]. Prevention of one episode of HAP may save 20,000$ [8].

Critical Illness Related Corticosteroid Insufficiency (CIRCI) reaches up to 50 to 70% of trauma patients [9-11]. CIRCI increases systemic inflammation and vasopressive requirement [9,12]. Hydrocortisone decreases rate of HAP and duration of mechanical ventilation in multiple trauma patients with CIRCI [10]. In a sub-group analysis of the HYPOLYTE trial, hydrocortisone appears particularly efficient in multiple trauma patients with TBI [10]. Fludrocortisone was proposed in association with hydrocortisone for the treatment of CIRCI in septic patients [13] and is recommended in BI patients with spontaneous subarachnoid hemorrhage who experience hyponatremia [14]. No data regarding fludrocortisone use in TBI patients are available to date.

The CORTI-TC study aims to test the effects of prolonged low dose of hydrocortisone together with fludrocortisone for prevention of HAP and long term neurological and psychological recovery in patients with severe TBI.

## Methods and Design

### Objectives and design

The Corticotherapy in traumatic brain-injured patients (CORTI-TC) study is a nationwide multicenter, randomized, double blind, placebo controlled trial. The Institutional Review Board of Tours (France) approved the study protocol. The CORTI-TC is conducted in accordance with the declaration of Helsinki and was registered on March 23 2010 at http://clinicaltrial.gov/ with trial registration NCT01093261.

### CONSORT diagram

**Figure 1:**
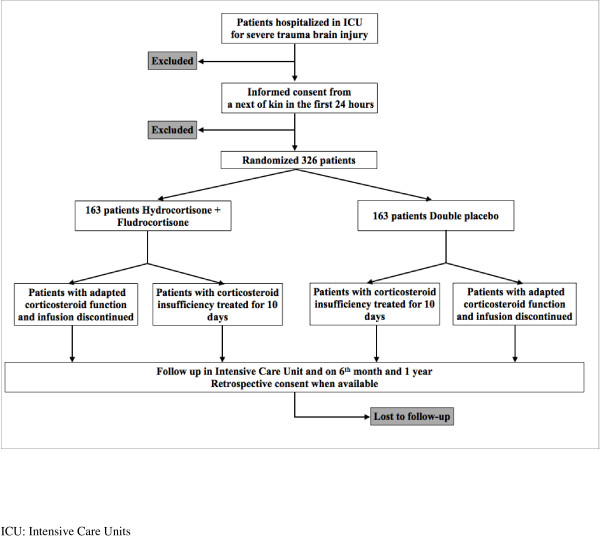
CONSORT diagram of the CORTI-TC trial

Figure 1 shows the CONSORT diagram of the CORTI-TC trial.

### Study population

Investigators screen consecutive severe TBI patients defined as the association of a Coma Glasgow Scale ≤ 8 together with a traumatic anomaly on tomodensitometry. Inclusions criteria are: severe traumatic BI, in the first 24 hours following trauma, age between 15 and 65 years and informed consent from a next-of-kin. Exclusions criteria are: associated tetraplegia, previous immunosuppression, corticotherapy in the previous 6 months, antibiotherapy for active sepsis at the time of inclusion.

### Randomization

Patients eligible for inclusion should be randomized, and the study treatment is started within the first 24 hours. Patients are randomized in a 1:1 ratio in fixed blocks of 12 and stratified according to the center and MGAP score [15] by a computerized number generator list provided by a statistician not involved in the determination of eligibility or in the assessment of outcomes. All assignments are made through a dedicated, pass-word protected, SSL-encrypted website. Randomized patients are given a number corresponding to a «Corti-TC treatment pack» that contains: 20 × 100 mg vial of hydrocortisone or placebo, 10 pills of 50 μg of fludrocortisone or placebo and a sheet for schedule administration.

### Study protocol

Immediately after randomization, and before study drug administration, a short corticotropin test is performed (basal cortisolemia followed by 0.25 mg of synacthene ^® ^and cortisolemia on the 60^th ^min.). Patients are randomized to intravenous infusion of hydrocortisone (200 mg.day^-1 ^for 7 days, 100 mg on days -8 and -9, 50 mg on day-10) with enteral administration of fludrocortisone (50 μg.day^-1 ^for ten days) or to placebo. After receiving the results of the short corticotropin test (usually in the first 48 hours following inclusion), the treatment is stopped if patients have an adapted corticosteroid function.

### Protocol drop-out

Clinicians are allowed to use corticotherapy at any time point if there is an absolute adrenal insufficiency (basal cortisolemia below 30 μg.dl^-1^), a septic shock or an Acute Respiratory Distress Syndrome. Enteral administration of the study drug (Fludrocortisone or Placebo) can be stopped in case of hypernatremia (>155 mmol.l^-1^) associated with a low natriuresis (< 20 mmol.l^-1^). The complete follow up is always performed and patients are kept in the statistical analysis.

### Biological assessment

Immediately before starting the treatment, but at least 8 hours after a bolus injection of etomidate [10,13], a short corticotropin test is performed: cortisolemia before and 60 minutes after an intravenous bolus of 0.25 mg of corticotropin (Novartis ^®^, Rueil-Malmaison, France). A second short corticotropin test is performed on day-11 or -12. At the same time points, plasma and serum are frozen at -80°C for ancillary studies.

### Study end points

Primary endpoint is the occurrence of HAP within 28 days of randomization. Pneumonia is considered when at least 2 signs (body temperature > 38°C; leukocytosis >12 000/mL, or leukopenia <4000/mL; purulent pulmonary secretions) associated with the appearance of a new infiltrate are present or when changes occur in an existing infiltrate on chest x-ray. The diagnosis needs to be confirmed by a respiratory tract sample using a quantitative culture with a predefined positive threshold of 10^4 ^colony-forming units per milliliter (CFU/mL) for a bronchoalveolar lavage or non bronchoscopic sample, of 10^3 ^CFU/mL for a protected specimen brush and of 10^6 ^CFU/mL for a tracheal sample. Hospital-acquired pneumonia is defined as pneumonia that occurs 48 hours after admission that had not been incubating at the time of admission.

The secondary outcomes are duration of mechanical ventilation and length of ICU stay, antibiotic free days, rate of death, other infections, organ failures, and duration of vasopressor support on day 28 and in ICU. Safety is assessed by recording adverse events.

An ancillary study will assess the mortality, the long term neurological and psychological recovery on the -6^th ^and -12^th ^month.

### Follow up

The following variables are collected: demographics, Apache 2, MGAP score [15], injury severity score and abbreviated injury score, fluid infusions, vasopressors, antibiotherapy, etomidate use, surgery, intracranial pressure, infections, organ failures, natremia, length of ventilatory support, and ICU hospitalisation and death in ICU and at day 28 are recorded. In ICU, clinical assessments are performed at least twice a day. Clinical evaluation for diagnosis of pneumonia are performed twice a day in the ICU. A chest x-ray is performed as soon as pneumonia is suspected after clinical examination. A respiratory tract sample is collected for bacteriological analysis before any modification of antibiotic therapy. Patients or relatives are contacted on -6 and -12 month for evaluation of neurological recovery (GOS, Kartz index), of quality of life, of depression disorder (HAD scale, SF-36 and I.E S-R scores) and of mortality.

### Statistical consideration

The required sample size is calculated from results of the HYPOLYTE study [10]. The power calculation is performed for comparison between the hydrocortisone and placebo groups in patients with corticosteroid insufficiency. The sample size needed to detect an absolute decrease in pneumonia incidence of 20% is 121 patients in each group, assuming a rate of 70% in the placebo group, in a 2-sided test performed with a statistical power of 90% and an α risk of 5%. Assuming a rate of 75% of corticosteroid insufficiency [10], 326 patients (approximately 163 patients in each group, and 41 patients with normal corticosteroid function who are untreated) are needed. Two a priori interim analyses using the O'Brien and Fleming method for α risk spending are planned respectively after the inclusion of 110 and 220 patients.

An ITT and a mITT analysis will be performed. Because the patients with adapted corticosteroid function are left untreated (when the cosyntropin test show no corticosteroid insufficiency), a modified intention-to-treat (mITT) analysis that includes only the patients with corticosteroid insufficiency is planned.

### Statistical analysis

Primary outcome is the occurrence of HAP within the first 28 days after randomization. The primary analysis will use a Cox multivariate proportional hazards model that includes 2 predefined covariable: MGAP [15] and Coma Glasgow Score. We will also perform complementary sensitivity analyses with an adjustement on etomidate use. Corresponding hazard ratios (HRs) along with their 95% confidence intervals (CIs) will be reported. The crude percentages and the Kaplan-Meier estimators will also be presented. Cumulative incidence will also be estimated for the primary outcome by treating deaths as competing risks. The Fine and Gray model [16] will be used. If a difference is confirmed for the primary endpoint, sub-group analysis for multiple trauma (presence or absence), hyporeactivity to adrenocorticotrophic hormone (presence or absence), early treatment administration (<18 hours after trauma or >18 hours) will be performed. In order to control the family wise error rate, a stepwise hierarchal testing procedure will be used for subgroup analyses.

Normally distributed variables will be expressed as mean and standard deviation; non-normally distributed variables will be expressed as median and inter quartile ranges. Categorical variables will be expressed as numbers and percentage, as well as the absolute difference (95% CI). Linear or logistic regressions will be used as appropriate for the secondary endpoints.

### Study Organization

The financial support are provided by the Société Française d'Anesthésie Réanimation (SFAR). Study promotion is performed by Nantes University Hospital.

The independent Data and Safety Monitoring Board (DSMB) look over the ethics in accordance with the Declaration of Helsinki, monitors patient safety and reviews safety issues as the study progresses. Serious adverses events and unexpected related or possibly related serious events are reported blinded to the DSMB within respectively 24 hours or 7 days.

Patients are treated in level I trauma centers according to the Advanced Trauma Life Support principles.

## Discussion

The Corti-TC trial is the second randomized controlled study powered to investigate prolonged low dose of corticosteroid therapy in severe trauma patients, and the first one focusing on severe TBI patients.

Cortisol production is necessary for normal host defence against infection. Hydrocortisone dampens inflammation while preserving innate immunity [17] and improves the recovery from severe community-acquired pneumonia [18]. Trauma-induced inflammation, which is exacerbated in cases of corticosteroid insufficiency [19], leads to infection [12]. In the HYPOLYTE study [10], low dose of hydrocortisone decreased rate of HAP in intubated trauma patients with corticosteroid insufficiency.

The primary outcome of this trial is HAP, that could be considered as a shortcoming since the prevention of HAP does not always improves outcomes of ICU patients [20]. Infections are the most frequent cause of complications in severely injured patients [21]. The incidence of pneumonia is particularly high in trauma patients, reaching up to 50% [2-4]. Post-trauma pneumonia is responsible for an increased morbidity (duration of mechanical ventilation and ICU hospitalization) [5,6], and the average cost for each pneumonia episode is high [8]. In the HYPOLYTE study, together with a decreased in rate of HAP, hydrocortisone decreased both duration of mechanical ventilation and ICU length of stay. Thus, we considered that the most relevant endpoint would be HAP.

Criteria for HAP diagnosis has not been studied in trauma patients treated with hydrocortisone and theoretically the study treatment may decrease the ability of clinician's to diagnose HAP. For community-acquired pneumonia, the body temperature of patients treated with a high dose of glucocorticoid was slightly decreased (by less than 0.5°C) as compared to placebo-treated patients [22], but to our knowledge, the effects of stress dose hydrocortisone (if any) are unknown in trauma patients. Also, in case of CIRCI hydrocortisone should restore the ability to develop fever, and patients without CIRCI (left untreated) should not present any dysregulation in thermoregulation abilities. Finally, we assumed that hydrocortisone with fludrocortisone will not biased HAP diagnosis.

Major modifiable risk factors for HAP as described by the American thoracic Society [23] are prospectively collected: oropharyngeal decontamination, proclive (>30°), stomach ulcer prevention, insulinotherapy, tracheostomy and protocol for sedation. All these factors can not be standardized in the protocol. Sedation is a cornerstone therapy for intracranial hypertension [24]. Therefore, it was not practical to perform either daily spontaneous breathing trials or daily liberation from sedation in a population of traumatic brain injury patients. Concerning tracheostomy, prevention of HAP by performing tracheostomy early is still debated. In a recent study, the incidence of HAP was similar in patients with early versus late tracheostomy [25]. None of the participating ICUs usually perform early tracheostomy or "daily spontaneous breathing" in TBI patients during the study period. Regarding insulinotherapy, tight glycemia control is a highly debated topic for ICU patients and is associated either with beneficial [26] or deleterious effects [27]. A tight glycemic control was not associated with any survival improvement of septic patients treated with hydrocortisone [28]. Proclive and oropharyngeal decontamination are commonly used in participating ICUs. Finally, a controlled double-blind randomized design may ensure the comparability of groups for the majority of interventions other than hydrocortisone infusion.

In this study, we use a placebo in a population of CIRCI patients. This point do not raise an ethical issue as the recommendations for the diagnosis and treatment of CIRCI recently published by the American College of Critical Care Medicine [29] state that: first, given the lack of data, treatment of CIRCI is not recommended in other population than septic shock patients; second, a call for studies in ICU populations other than septic patients (including trauma patients) was advocated. Moreover, a placebo is ethically justified because the placebo is given in addition to standard of care of TBI patients.

The selection of ICU patients who require hydrocortisone treatment is of particular importance. The concept of CIRCI has recently been introduced to differentiate the classical chronic corticosteroid insufficiency (Addison's disease) from the alteration of the hypothalamic-pituitary-adrenal axis observed in ICU patients [29]. CIRCI corresponds to an inadequacy between the severity of illness and the corticosteroid activity [19]. The combination of basal cortisolemia and/or a delta <9 μg/dl provides the best specificity and sensitivity for the diagnosis of CIRCI associated with severe sepsis [30]. Finally, recent recommendations have advocated use of either the basal cortisolemia or delta for diagnosis of CIRCI [31]. Regarding threshold of the basal cortisolemia, our definition is more liberal (<15 μg/dl) than the consensus statement (< 10 μg/dl). In the HYPOLYTE study using this liberal definition [10], patients with a normal corticosteroid function have a trend toward a decrease in rate of HAP as compared to patients with corticosteroid insufficiency treated with placebo (P = .06). Finally, the Corti-TC trial is not designed to test the accuracy of the CIRCI definition in trauma patients.

Despite inhibition of the cortisol synthesis, etomidate is recommended for Rapid Sequence Induction of multiple trauma patients [31]. For single bolus injection, the duration of adrenal blockade varies from 12 hours [32] to 24 hours [33], but it should be noted that this blockade was inconstantly reported [34]. In the Corti-TC study, a corticotropin test will not be performed within the first 8 hours following etomidate infusion and etomidate use in ICU is discouraged. Finally, as etomidate use may impact the results, etomidate will be considered as an effect modifier for the analysis (sensitivity analysis).

In conclusion, the Corti-TC trial is a nationwide investigator-initiated randomized controlled trial powered to test the hypothesis that the association of hydrocortisone with fludrocortisone in severe TBI patients prevents HAP. The Corti-TC trial also determines the effects of low dose of corticosteroids on duration of mechanical ventilation, length of ICU stay, organ failures and rate of death. An ancillary study will assess the long term neurological and psychological recovery of the included patients.

## Trial status

On October, the 5th of 2011, 110 patients have been enrolled in the study. The first interim analysis will be performed in november 2011, after the collection of the primary end point of the 110th patient.

## List of abbreviations

BI: Brain Injury; CIRCI: Critical Illness Related Corticosteroid Insufficiency; CFU: Colony Forming Unit; DSMB: Data and Safety Monitoring Board; HAP: Hospital Acquired Pneumonia; ICU: Intensive Care Unit; ITT: Intention-to-treat.

## Competing interests

The authors declare that they have no competing interests.

## Authors' contributions

KA, AR: preparation of the initial drafts of the manuscript and preparation of the final version. The corti-TC trial group: review of the initial drafts of the manuscript. VS: planned the statistical analysis and revised the manuscript. KA, AR, VS: designed the study, reviewed the initial drafts of the manuscript. All authors approved the final version of the manuscript.
